# Does personality still matter in e-commerce? How perceived hubris influences the assessment of founders’ trustworthiness using the example of reward-based crowdfunding

**DOI:** 10.1007/s12525-022-00584-6

**Published:** 2022-09-14

**Authors:** Janina Sundermeier, Tyge-F. Kummer

**Affiliations:** 1 Department of Information Systems, Freie Universität Berlin, Garystrasse 21, 14195 Berlin, Germany; 2 Queensland University of Technology, QUT Business School, 2 George Street, 4000 Brisbane, Queensland Australia

**Keywords:** Reward-based crowdfunding, Personality traits, Hubris, Survey, Intuitive information processing, D81, M13

## Abstract

Reward-based crowdfunding broadens the scope of e-commerce transactions, as prototypical products are pre-sold under conditions of considerable uncertainty. To date, we know little about the mechanisms that underlie decisions to back reward-based crowdfunding campaigns. However, it is likely that startup founders’ possibility of showcasing their personalities in video pitches signals their trustworthiness, particularly, as other features, such as quality seals and customer testimonials, are often unavailable. We use signaling theory to reinforce the move from a feature-oriented perspective to a signaling perspective, as signals can transmit information about startup founders’ otherwise imperceptible qualities and abilities. Based on a survey (*N* = 108), we investigate how perceived hubris – proven to be particularly salient in startup contexts – influences the funding decision of potential backers. We find that abilities and legitimacy of a startup founder are rated positively when s/he is perceived as hubristic. These results have implications for crowdfunding campaigns and highlight the relevance of personality traits in electronic markets.

## Introduction

E-commerce has grown continuously over the last decade. Additionally, its growths accelerated substantially during the Covid 19 pandemic, which confined large portions of the world’s population to their homes and led to repeated retail store closures. In fact, the retail volume of non-food products in Europe has dropped by 11.6% in April 2020 (Eurostat, 2022), while the share of e-commerce transactions increased by an average of 10% and even exceeded the share of retail transactions during the multiple lockdowns (Statista, 2021). Also crowdfunding has been used during this time to either secure one’s existence or to meaningfully use time at home to advance one’s business (Igra et al., 2021). The comparability of underlying mechanisms has led to reward-based crowdfunding being increasingly associated as a form of e-commerce where, among other benefits, backers are offered the opportunity to pre-purchase a product as a reward for their investment (Belleflamme et al., 2014; Bi et al., 2017; Cholakova & Clarysse, 2015). Sourcing a future product’s costs from a large crowd of small, non-professional investors, referred to as backers or crowdfunders, is particularly popular with startup founders aiming to overcome their limited financial means (Kunz et al., 2017; Schwienbacher & Larralde, 2012; Thies et al., 2018). However, backers who choose to pre-purchase future products in return for their investments are “subject to an unusually high degree of risk” (Agrawal et al., 2014, p. 68) because of considerable information asymmetries (Ahlers et al., 2015; Burtch et al., 2013; Kuppuswamy & Bayus, 2018) that arise from the lack of reliable information with regard to the product’s characteristics, the startup founder’s identity, and the ability to enforce contracts that are concluded online (Datta & Chatterjee, 2008; Granados et al., 2006; Short et al., 2017). The e-commerce literature posits that trust built through a variety of features is imperative to mitigate such information asymmetries (Guo et al., 2018; Kim & Peterson, 2017).

However, transferring existing theory about efficient trust-building determinants in regular online commerce, which typically offers well-established goods and services, to the pre-selling of innovative products in reward-based crowdfunding contexts has limitations. In particular, crucial features, such as customer testimonials and quality seals (Everard & Galleta, 2006; Jarvenpaa et al., 2000; Yoon & Occeña, 2015), that have been shown to be efficient to build trust in regular online retailing transactions, are seldom available for startup founders, whose businesses and products are largely unknown (Bi et al., 2017; Burtch et al., 2013; Cholakova & Clarysse, 2015). In addition, customers must be convinced that the product will be successfully produced, which requires an assessment of the company’s capabilities and potential market demand. In an attempt to provide alternative explanations for what determines trust in this form of e-commerce transaction, the focus of research on crowdfunding is increasingly shifting from a feature-oriented perspective to a signaling perspective (Ahlers et al., 2015; Allison et al., 2013; Courtney et al., 2017; Moss et al., 2015; Steigenberger & Wilhelm, 2018). Signals are verbal and non-verbal cues that transmit relevant information that backers consciously or unconsciously process in assessing startup founders’ ambitions, capacities, and skills (Connelly et al., 2011; Kunz et al., 2017). Especially the personality of entrepreneurs (OED, 2015) - a combination of characteristics or qualities that form an individual’s distinctive character – and their various personality traits, defined as enduring propensities to act in certain ways, are found to serve as signals that allow to assess an individual’s behavior (Allport, 1961; McElroy et al., 2007; Rauch & Frese, 2007). Research to date has shown that personality is highly relevant in day-to-day business activities as signaling a disposition to innovation, autonomy (Allison et al., 2013), competitiveness aggressiveness, and risk-taking (Moss et al., 2015), as well as agreeableness and openness (Thies et al., 2016), positively influence the outcomes of online investment decisions. To further advance the state of knowledge in these regards, we aim to establish a link between personality traits and trustworthiness – the key determinant of successful e-commerce transactions (Guo et al., 2018; Kim & Peterson, 2017) – by examining how personality serves as a signal for trustworthiness in reward-based crowdfunding campaigns.

To achieve this objective, we focus on perceived hubris, a comparatively extreme personality dimension that is characterized by excessive pride, exaggerated confidence, and an inflated feeling of self-worth (Judge et al., 2009; Owen & Davidson, 2009; Petit & Bollaert, 2012), based on the following reasons. First, the hubris theory of entrepreneurship suggests that these traits are particularly salient in startup contexts, as hubristic startup founders in particular are well equipped to handle high levels of uncertainty, time constraints, and considerable failure rates (Bollaert & Petit, 2010; Hayward et al., 2006; Ranft & O’Neill, 2001). Second, the focus on specific and clearly delineated traits is shown to have a higher predictive validity compared to broader traits, such as the ‘big five model’ (Aldrich, 1999; Barrick & Mount, 2005), which has been widely used in previous research. Third, recent evidence indicates an interesting ambiguity between the positioning of hubris as a ‘dark’ personality dimension and hubristic founders’ potential to signal a strong ability to turn the business into a success story, that is not yet well understood (Sundermeier et al., 2020).

Reward-based crowdfunding campaigns provide a unique context to examine the role of backers’ perceptions of startup founders’ personalities, given the influence of video pitches on the success of such campaigns (Kickstarter, 2021; Mollick, 2014). To generate empirical evidence for the relevance of hubris for the outcomes of reward-based crowdfunding campaigns, we conduct a survey (*N* = 108) by drawing upon an US MTurk sample to examine these relationships empirically. This methodological approach allows us to provide answers to the following research question: *How does perceived hubris influence startup founders’ trustworthiness in reward-based crowdfunding campaigns?*

By answering this question, the paper contributes to the literature on the determinants of funding intention in reward-based crowdfunding scenarios. Our findings indicate that in the absence of trust-building determinants that have been shown to be effective in regular online retailing transactions, personality traits such as hubris are powerful signals that allow startup founders to mitigate information asymmetries and thereby influence the investment intentions of potential backers. By drawing on signaling theory, we show that the personality displayed in a pitch video serves as a signal for the assessment of startup founders’ trustworthiness. To that end, we observe that the perception of hubris activates specific trust dimensions, such as legitimacy and ability, while other factors such as benevolence and empathy occur only when hubris is not perceived. With these findings, we also contribute to the hubris theory of entrepreneurship in the sense that we provide empirical evidence that hubris should not be viewed as an exclusively dark personality dimension leading to suboptimal behavior, as claimed in some recent publications on hubristic leadership (Sundermeier et al., 2020; Tang et al., 2018). On the contrary, their personalities signal strong abilities and legitimacy, allowing them to reduce information asymmetries that would otherwise discourage crowdfunders from completing the online transaction.

We proceed as follows: After we present the state of the extant literature on what determines crowdfunders’ perceptions of trustworthiness, we introduce hubris and, drawing on signaling theory, discuss the relevance for the assessment of startup founders’ trustworthiness. Next, we describe the selected methodology and present the results. After discussing the results, we conclude with a discussion of theoretical and practical implications.

## Theoretical background

Unlike equity crowdfunding, backers who pledge money in reward-based crowdfunding campaigns are offered non-monetary rewards like prominent credit for the final product, involvement in the creative product-development process, a meeting with the startup founders, a copy of the product, or the option to pre-purchase the product (Bi et al., 2017; Cholakova & Clarysse, 2015). A prominent example of such a campaign is the smartwatch Pebble, for which 68,929 crowdfunders pledged more than $10 M in return for the first model (Agrawal et al., 2014; Brown et al., 2017; Mollick, 2014). The option to pre-purchase a tangible product in return for an investment makes funders early customers and triggers the association of reward-based crowdfunding as a form of e-commerce (Ahlers et al., 2015; Beier & Wagner, 2015; Gierczak et al., 2014). The initiators of Kickstarter, a leading platform for reward-based crowdfunding campaigns, deny this analogy by emphasizing that “many people feel like they’re shopping at a store when they’re backing projects on Kickstarter, but we want to make sure that it’s not one” (Strickler et al., 2012). The essence of this quote refers to the distinct scopes of regular online retailing and reward-based crowdfunding. Regular online retailing is pursued to sell, trade, and distribute products and services that already exist (Chiu et al., 2014; Gefen et al., 2003; Guo et al., 2018), while the usual objective of reward-based crowdfunding is to source money for making new products that do not yet exist (Burtch et al., 2013). The novelty of the product and the limited reputation of the startup founders who pursue such campaigns make it difficult for them to show that they are trustworthy and capable of successfully manufacturing the products described in their campaigns.

## Determinants of trustworthiness in e-commerce and reward-based crowdfunding

The e-commerce literature shows that trustworthiness acts as an informal control mechanism that reduces friction, limits opportunistic behaviors, minimizes the need for bureaucratic structures, and helps build long-term relationships (Bhattacherjee, 2002; Fang et al., 2014; Kim & Peterson, 2017). A lack of trust is “one of the greatest barriers inhibiting internet transactions” (Kim et al., 2004, p. 393), and buyers are willing to pay price premiums when they perceive online retailers as trustworthy (Ba & Pavlou, 2002; Gefen et al., 2003; D. Kim & Benbasat, 2009; Kim, 2014). Buyers’ trust is decisively influenced by how confident they are with respect to sellers’ *ability*, *benevolence*, and *integrity* (Fang et al., 2014; Mayer et al., 1995). These three dimensions are core cognitive and affective elements that determine trust formation in many contexts (G. Jones & George, 1998; Kim et al., 2004; Singh & Sirdeshmukh, 2000). The rising relevance of signals including all kinds of visual cues for the successful complementation of e-commerce transactions has prompted several scholars to additionally include *empathy* and *legitimacy* in their assessment of online retailers’ trustworthiness (Fairchild, 2011; Kwak et al., 2019).

For startup founders, the *ability dimension* refers to their competencies, skills and knowledge (Gefen et al., 2003; Guo et al., 2018). Backers who support reward-based crowdfunding campaigns need to assess whether the founder has the necessary abilities to convey the prototypical product into a marketable good (Burtch et al., 2013; Courtney et al., 2017; Zhang & Liu, 2012). The *benevolence dimension* refers to the extent to which the startup founders have good intentions beyond their own profit (Fang et al., 2014) and are *empathic* to understand the need and wants of others. Backers need to assess whether the founder is receptive to the target group’s needs so people feel addressed and pledge the money that will meet the founder’s funding goal (Ahlers et al., 2015; Colombo et al., 2015). The *integrity dimension* refers to the extent to which the founder is expected to adhere to a set of principles or rules of exchange that is acceptable to all parties involved (Bhattacherjee, 2002; Kim & Peterson, 2017), which is closely linked to their *legitimacy,* describing the extent to which their actions taken to turn the envisioned product into reality are perceived as desirable and appropriate. To that end, backers need to assess to whether the founder is willing to be fair in conducting the transaction by producing a product that carries the promised values and is delivered on time (Herzenstein et al., 2011; Mollick, 2014).

Features that enhance buyers’ trust in regular online retailing transactions, such as customer testimonials and direct communication channels (Jarvenpaa et al., 2000; Yoon & Occeña, 2015), design features and quality seals (Everard & Galleta, 2006; K. Jones & Leonard, 2008; Schlosser et al., 2006), and various security measures (Hu et al., 2010; Kim et al., 2004; McKnight et al., 2002), are seldom available to startup founders. In particular, first-time founders do not usually have any kind of reputation, cannot offer any credible customer testimonials for a product that does not yet exist, and usually operate on third-party platforms whose design features they can hardly influence (Bi et al., 2017; Cholakova & Clarysse, 2015; Kim et al., 2008). Potential backers can assess information related to the future product’s characteristics and features only through textual and visual descriptions provided by a single source, the founders themselves (Efrat & Gilboa, 2020; Schwienbacher & Larralde, 2012; Thies et al., 2016). However, videos can significantly increase the chances of achieving the funding target (Mollick, 2014).

To find alternative determinants that can influence crowdfunders’ investment intentions, scholars examine the suitability of diverse signals to transmit intangible information regarding the characteristics of the founders and their ventures (Ahlers et al., 2015; Allison et al., 2013; Moss et al., 2015; Steigenberger & Wilhelm, 2018). Their findings indicate that the number of previous backers and their assessments of campaigns, as shared through their social networks, are quality signals that can influence crowdfunders’ investment decisions (Herzenstein et al., 2011; Lin et al., 2009; Thies et al., 2018; Zhang & Liu, 2012). However, these insights explain only network effects, not how the network of supporters grew in the first place or how the early backers’ perceptions of startup founders’ trustworthiness was influenced, even though it appears that perceptions of certain personality traits of startup founders play a central role in this regard (Bollaert et al., 2020; Sundermeier & Kummer, 2018; Thies et al., 2016).

## Signaling hubris to be perceived as trustworthy

Personality traits are enduring propensities to act and exhibit certain types of responses to situations (Caprana & Cervone, 2000; Devaraj et al., 2008). Thus, they are considered suitable predictors of an individual’s behavior (McElroy et al., 2007; Rauch & Frese, 2007). Given the frequency of video pitches in which founders, their products, and their ventures appear in crowdfunding campaigns (Mollick, 2014), scholars have started to focus on the impact of personality traits on crowdfunders’ investment intentions (Ahlers et al., 2015; Allison et al., 2013; Moss et al., 2015). This research interest is triggered by studies in traditional offline investment scenarios that determine that startup founders’ personality traits influence venture capitalists’ and business angels’ investment decisions (Cardon et al., 2009; Chen et al., 2009; Sudek, 2007). However, these studies observe the effects during direct interactions between founders and investors. The extent to which personality traits or other cognitive features influence investment decisions in online contexts (without personal interaction) remains unclear (Hoegen et al., 2018). The first empirical findings in this regard indicate that showing an entrepreneurial orientation and the ‘big five’ personality dimensions positively influences backers’ investment intentions (Allison et al., 2015; Moss et al., 2015; Thies et al., 2016).

These contributions provide a valuable step forward in theory development on the determinants of crowdfunding outcomes, but their broad scope carries risks that led management scholars to conclude two decades ago that “research on personality traits seems to have reached an empirical dead end” (Aldrich, 1999, p. 76). This criticism was directed towards broad personality traits like the ‘big five’ model to describe the human personality and psyche (Cogliser & Brigham, 2004; Ensley et al., 2006; Rauch & Frese, 2007). Studies that use these broader traits are significantly lower in their predictive validity than studies that focus on single, narrow traits and “rely on explicit descriptions that may be situated in time, place, or role” (Barrick & Mount, 2005, p. 367). One of such narrower traits that is directly linked to venture creation processes is hubris (Bollaert & Petit, 2010; Picone et al., 2014; Ranft & O’Neill, 2001). The hubris theory of entrepreneurship suggests that hubris is particularly salient in startup contexts, as it supports founders’ abilities to enact their seemingly far-fetched plans despite high failure rates, time constraints, and high levels of uncertainty (Hayward et al., 2006). The term *hubris* originates from Greek mythology and describes a set of several personality traits, such as excessive pride, exaggerated confidence, and an inflated feeling of self-worth (Judge et al., 2009; Owen & Davidson, 2009; Petit & Bollaert, 2012). Previous studies indicate, that despite being labelled a dark personality trait, hubris is likely to have ‘bright’ outcomes for venture performance, as hubristic leaders may have a strong vision that they pursue without being frightened by challenges (Judge et al., 2009; Sundermeier et al., 2020; Zuckerman & O’Loughlin, 2006). Haynes et al. (2015) are the first to discuss conceptually the influence of hubris on perceptions of entrepreneurs’ trustworthiness, but empirical findings in this regard are scarce.

In order to understand how the perception of hubris influences investment decisions communication models can be used. Traditional approaches such as the transmission model (Shannon, 1948) understand communication as the transmission of information between a sender and a receiver via a certain channel. While these models are highly influential in communication studies, they do not consider interpretation differences on the receiver side. The semiotic theory overcomes this problem as it assumes that “[…] messages are made of signs and conveyed through sign systems called codes; meaning is derived only to the degree that the receiver of the message understands the code.” (Moriarty, 2002, p. 22). A sign is defined as an element that has meaning for someone in some respect or capacity (Peirce, 1991). However, there is a difference between what we mean, what we say, and how it is perceived from a particular perspective in a specific context (Chandler, 2017). This theoretical frame shows similarities with signaling theory, suggesting that signals can reduce information asymmetries between two parties (Janney & Folta, 2003; Spence, 1973). The information that the sender provides can be interpreted as signals that are not readily available to others and influence the decision making of the receivers (Connelly et al., 2011; Spence, 2002; Stiglitz, 2000).

In the context of crowdfunding, startup founders are the signal senders as they have intimate knowledge about the venture’s prospects, their commitments, and the state of product development. Backers are the receivers of these signals as they need this information to make informed inferences about whether they should commit a financial investment or not (Ahlers et al., 2015; Busenitz et al., 2005). To that end, founders are eager to meet this need by communicating the knowledge they have so potential backers will decide in their favor (Ahlstrom & Bruton, 2006; Mäkelä & Maula, 2006; Schwienbacher, 2007). Using the lens of semiotics, we explore how the personality trait of hubris and the verbal and non-verbal communication associated with this perceived trait influences potential investors. However, since the perception of signs is subjective, we differentiate between those potential backers who perceive the specific personality trait of hubris and those who do not in order to determine differences in the underlying mechanisms that build trustworthiness and influence investor’s decision making.

## Research model

We examine how perceptions of hubris influence the funding intention of potential backers in reward-based crowdfunding scenarios. Therefore, we determine how the perception of hubris signals trustworthiness, which we capture through five different dimensions. Furthermore, we investigate how these dimensions of trustworthiness influence the expected product success, which captures the extent to which potential backers believe that the product will be successful as well as the intention to back the crowdfunding campaign to obtain the product.

## Operationalization of hubris

The scholars adhere to established conceptualizations (Judge et al., 2009; Owen & Davidson, 2009; Petit & Bollaert, 2012) and define (perceived) hubris as a personality dimension that is characterized by excessive pride, exaggerated confidence, and an inflated self-worth. People with high levels of confidence tend to overestimate their self-worth, talents, abilities, and accomplishments (Hiller & Hambrick, 2005; Judge et al., 2009), which strengthens their belief in own abilities to succeed and leads them to engage in risky endeavours (Hayward & Hambrick, 1997; Owen & Davidson, 2009; Petit & Bollaert, 2012). Case studies of hubristic startup founders like Mark Zuckerberg and Jeff Bezos indicate that the expression of these traits projects power, strength, and authority (Bollaert & Petit, 2010; Judge et al., 2009) and positively affects external actors’ perceptions (Hayward, 2007; Ranft & O’Neill, 2001). Such founders are seen as the main drivers of innovation processes, as they signal that they have sufficient courage and an unshakable belief in their ability to initiate new ventures successfully (Hayward et al., 2006; Picone et al., 2014). On the downside, excessive pride, which, unlike authentic pride, is often associated with arrogance, can negatively influence perceptions of an individual’s authenticity (Tracy et al., 2009). Hubristic founders tend to focus primarily on self-enhancing values and attribute positive achievements to themselves (e.g., “I am successful because of my intelligence”) (Haynes et al., 2015). The public expression of their superiority can signal aggression and a negative view of others, which are antisocial tendencies that provoke dislike (Hoorens et al., 2012; Van Damme et al., 2016).

## Influence of perceived hubris on crowdfunders’ funding decisions

Building on recent findings on hubristic leadership (Sundermeier et al., 2020; Tang et al., 2018), we argue that hubris signals both beneficial and detrimental attributes depending on the activity for which hubristic founders are evaluated. Their visionary power and unwavering belief in their value proposition is likely to signal strong abilities, integrity, and legitimacy to turn the promised product into reality. Yet hubristic founders are highly self-centered and focused on their own needs and desires, which might impair their ability to signal benevolence and empathy for the desires of others. In particular, their exaggerated confidence and lack of self-doubt convinces hubristic entrepreneurs that their products and services have the potential to disrupt existing markets (Sundermeier et al., 2020), which might not necessarily be the case in reality (Picone et al., 2014; Ranft & O’Neill, 2001). In more detail, expressing their unshakable belief in themselves and their products signals power, strength and authority (Judge et al., 2009; Owen & Davidson, 2009), attributes that are frequently associated with ability (Hayward et al., 2006; Thies et al., 2016). Using these findings, we argue that hubris is a strong signal of the competencies and skills that are required to convey a prototypical product to the market and to handle the transactions necessary to manufacture and deliver the final product (Sundermeier et al., 2020). The perception of such abilities is likely to be one of the core drivers of crowdfunders’ expected product success. This is highly relevant in a crowdfunding setting as receiving the promised product depends on the founder’s ability to meet the funding target and produce the product as promised (Burtch et al., 2013; Kuppuswamy & Bayus, 2018; Thies et al., 2014). Therefore, we argue that hubris serves as a signal for a startup founder’s perceived abilities, which positively influences crowdfunders’ expectation that the product will be successful. We hence state:

- *H1: In case the entrepreneur is perceived as hubristic, the perceived ability of the entrepreneur will positively influence the investment decision.*

Similar to the ability dimension, the startup founders’ integrity is likely to be positively influenced by their perceived hubris signaling that they have the power and strength required to fulfill the online transaction involving the delivery of the final product as described in their video pitch (Claxton et al., 2015; Hayward & Hambrick, 1997; Picone et al., 2014). The signal transports their ambition and willingness to establish target-oriented collaborations with partners necessary to manufacture and deliver the future product. To that end, their overly self-confident appearance is discussed to leave no doubt that they will do everything possible to fulfil the terms and conditions of the contract between them and the backers (Eckhaus & Sheaffer, 2018). Therefore, we propose that perceived hubris serves as a signal that positively influences the assessment of startup founders’ (perceived) integrity, which has positive implications for the expected product success:

- *H2: In case the entrepreneur is perceived as hubristic, the perceived integrity of the entrepreneur will positively influence the investment decision.*

Closely related to their perceived integrity is their legitimacy, as it is discussed that hubristic founders convincingly signal that they retain control over all necessary activities and the progress of their projects (Brady & Davies, 2010; Kroll et al., 2000; McManus, 2016). This signal is of particular importance in reward-based crowdfunding scenarios since backers pre-purchase a future product and need to trust the legitimacy of the founder to be able to turn the vision into an actual product that meets the values communicated in their pitch. Their overly self-confident appearance is hence expected to be perceived as a signal that hubristic founders neither spare any efforts to fulfill their side of the contracts nor their promise to deliver the intended product as presented. Following this line of argument, we state:

- *H3: In case the entrepreneur is perceived as hubristic, the perceived legitimacy of the entrepreneur will positively influence the investment decision.*

On the downside, hubristic founders are also highly egocentric as expressed through their strong focus on self-enhancing values (Haynes et al., 2015; Hoorens et al., 2012; Van Damme et al., 2016). As a consequence, they often fail to take other parties’ opinions into consideration (Brady & Davies, 2010; Kroll et al., 2000; McManus, 2016). This approach is especially problematic for startup founders, who usually set out to develop a value proposition that addresses the needs and wants of a broad target group. Since hubristic founders hardly consider needs and wants beyond their own, they are unlikely to validate their value proposition through interactions with their target group (Picone et al., 2014; Ranft & O’Neill, 2001). Since potential backers, however, assess the extent to which startup founders are receptive to their needs and willing to address them in the future product (Ahlers et al., 2015; Colombo et al., 2015), we argue that backers rate hubristic founders’ benevolence as low. Nevertheless, a positive assessment of benevolence is expected to be another determinant of backers’ expected product success, as they can expect a future product that meets their expectation only if the product is backed by many people who believe that the product can be produced successfully*.* Consequently, we propose that signals of hubris are counterproductive for the assessment of a startup founders’ (perceived) benevolence, which is why only:

- *H4: In case the entrepreneur is not perceived as hubristic, the perceived benevolence of the entrepreneur will positively influence the investment decision.*

As with benevolence, we expect hubristic founders to be perceived as non-empathic, as they focus on their own needs and wants instead of those of their target group (Hayward et al., 2006; Ranft & O’Neill, 2001). Hence, we argue that hubristic entrepreneurs cannot signal convincingly that they are empathic to the needs of their target group, although (perceived) empathy is expected to have a positive influence on crowdfunders’ expected product success.

- *H5: In case the entrepreneur is not perceived as hubristic, the perceived empathy of the entrepreneur will positively influence the investment decision.*

Extant studies show that the expected product success is a determinant of potential backers’ purchase intentions (Bi et al., 2017; Cholakova & Clarysse, 2015), so we hypothesize:

- *H6: The expected product success positively influences the crowdfunders’ intention to support the campaign.*

**Fig. 1:**
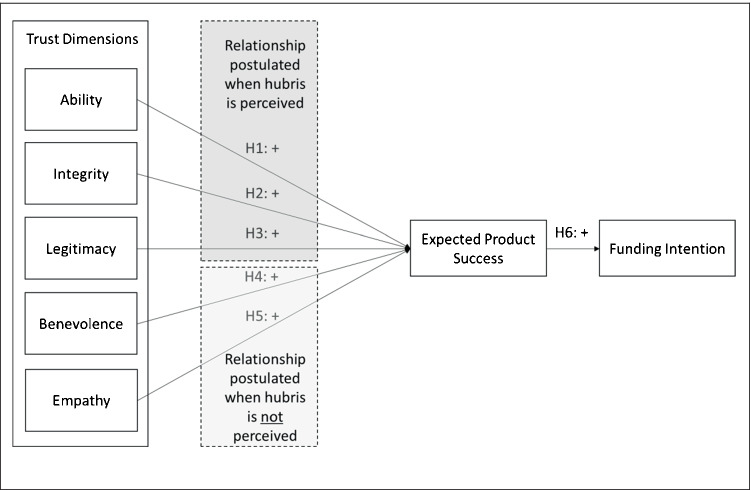
Research model – The influence of hubris on the funding intention when hubris is perceived or not perceived

Figure 1 summarizes the research model.

## Methodology

The research design combines a video pitch with a survey. The video expressed hubris to measure how its perception affects the facets of perceived trustworthiness that encourage backers to invest in reward-based crowdfunding campaigns.

## Materials

The survey contained a pitch video that was adapted from a real-world crowdfunding campaign. The campaign was selected based on four criteria:

1. *The founder succeeded in raising the targeted amount of money*
  To ensure that the product featured in the video is of interest to a wider audience, and thus that there is an actual market for the product, we focused on campaigns that have been successfully completed.
2. *The product and pitch are easy to understand without specific knowledge*
  To avoid biases resulting from misunderstandings about the features of the core product and the value it brings to customers, we screened campaigns for easy-to-understand products that have the potential to solve a problem that is familiar to a broader audience.
3. *The product price is relatively low and affordable by most*
  We also wanted a campaign that offered an affordable product to minimize biases due to limited financial resources that could further influence the investment decision.
4. *The product is still relatively unknown and not yet established in the market*
  To avoid biases based on the reputation a product already enjoys, we further narrowed down the number of campaigns that already meet the aforementioned selection criteria to those that have not received significant (social) media attention.

To identify appropriate pitches, we considered successful crowdfunding campaigns across leading platforms that feature reward-based crowdfunding campaigns, such as Kickstarter and IndieGoGo. We selected a non-absorbent T-shirt^1^ as the campaign best fulfilled all our criteria. Next, we examined the arguments about the T-shirt’s benefits that were presented in the original pitch. Then, based on a literature analysis that indicated that hubristic individuals tend to talk fast and snappy, with a contemptuous inflection and a focus on themselves and their personal attributes, we determined the video’s ‘tone and temper’ in terms of the personality traits that are associated with hubris (Bass, 1990; Baumeister et al., 2003; Todorovic & Schlosser, 2007). These characteristic expressions of hubris were discussed with a professional native English-speaking actor and a director to determine how they would be most naturally expressed in a pitch video.

Since we intend to develop a model that explains crowdfunding success based on hubristic personality traits, we selected a presentation style that displayed hubris characteristics, including a focus on the founder (e.g., “I can change something”) and the founder’s vision, signaling exaggerated confidence and excessive pride. The first version of the pitch was recorded and discussed with colleagues, founders, and supporters of crowdfunding campaigns to ensure the video pitch was realistic. Feedback from this discussion was used to rework the scripts, and the final pitch was recorded with a professional actor using a university’s professional video-recording facilities.

The survey began with an explanation of the scenario and the video pitch, followed by the question items to measure the constructs in our research model. In the last part of the survey, demographic data was collected, including age, gender, and experience with crowdfunding. All constructs were measured on a 7-point Likert-type scale, as suggested in previous research from which we adopted our items and scales. Appendix Table 3 contains all question items related to the latent variables in the research model.

Since the perception of hubris is subjective, we used three question items addressing different facets of hubris: excessive self-confidence, exaggerated pride, and inflated positive self-evaluations to calculate a formative latent variable score using SmartPLS 3. Based on this score, a median split was conducted (Iacobucci et al., 2015; Rhodes et al., 2002). The reasoning behind that split is that hubris is an extreme trait, and it is unlikely that the relationship between hubris and trust is linear. Therefore, we separate aspects such as ‘normal’ self-confidence, which is to some degree expected from an entrepreneur, from excessive self-confidence.

In order to test our hypotheses, we conducted a structural equation model estimation using partial least squares (PLS-SEM) (Hair et al., 2013). The PLS analysis was performed for each group (high and low split) independently and an additional PLS multi-group analysis (MGA) was used to compare both groups directly in a post hoc analysis.

## Participants

The data collection took place in February 2021. 108 Participants were recruited via Amazon MTurk with a filter setting to include only US residents to minimize the influence of external factors (e.g., the popularity of crowdfunding in different countries). Based on a moderate effect size (0.15) and an α error probability of 0.05, G*Power 3.1 suggests a statistical power greater than the recommended 0.80 for that sample size (Faul et al., 2007). Table 1 contains the demographic data of the participants. For the median split, the sample was divided into two subgroups (*N* = 64 and *N* = 46). Since hubris is an extreme behavior, we assigned the median to the group that does not perceive hubris. Thus, the group sizes differ. The hubris perception differs substantially between the groups. While the mean in the high split group is 6.07 (out of 7), it is only 3.34 in the low split group. The educational level of the participants was high as more than 62% had completed some form of higher education (Bachelor, Master, or PhD).

**Table 1:** Participants’ demographics for the entire sample

Participants (completed questionnaires)	108
Gender	
Female	39.81%
Male	60.19%
Not specified	0.93%
Age (mean) in years	42.85
Highest eeducation	
No school completed High school graduate, diploma or equivalent Trade/technical/vocational training Bachelor’s degree Master’s degree Professional degree Doctorate degree	0.00% 16.67% 19.44% 54.63% 6.48% 0.93% 1.85%
Perceived hubris (7-point Likert Scale)	
High split group (mean) Low split group (mean)	6.07 3.34

## Results

### Measurement model

We evaluate the measurement model quality using indicator reliability, internal consistency, as well as convergent and discriminant validity (Chin, 2010; Hair et al., 2012). We measured the *indicator reliability* based on the factor loadings. According to Chin (2010), all factor loadings should be above the threshold of 0.7. This criterion is fulfilled in both groups (high and low median split; see Appendix Table 3). *Internal consistency* can be determined by calculating Cronbach’s alpha and composite reliability (Hair et al., 2012). Cronbach’s alpha of all constructs in both groups is greater 0.8, and therefore above the recommended threshold of 0.7. Similarly, *composite reliability* is always above 0.8 suggesting adequate internal consistency. Moreover, the average variance extracted (AVE) is for all constructs above 0.7, fulfilling the criteria for *convergent validity* (Bagozzi & Yi, 1988). All constructs load primarily on the related construct (factor loadings on the main construct exceed cross-loadings, see Appendix Tables 4 and 5), and the Fornell–Larcker criterion is fulfilled (square root of the AVE scores exceed construct correlations, see Appendix Tables 6 and 7) (Fornell & Larcker, 1981). We conclude that *discriminant validity* is achieved.

### Structural model

As expected for the group in which hubris was perceived, a positive relationship emerges between *ability* and *expected product success* (path coefficient = 0.36, *p* < = 0.05), and between *legitimacy* and *expected product success* (path coefficient = 0.67, *p* < = 0.001). H1 and H3 are supported. However, we do not find support for a positive relationship between *integrity* and *expected product success.* Instead, a non-significant negative relationship emerged (path coefficient = −0.19, *p* > 0.1).

**Fig. 2:**
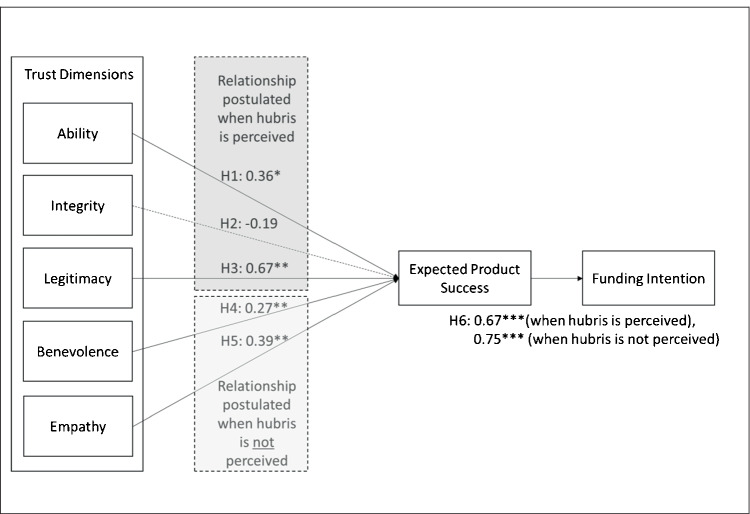
Structural model when hubris is perceived or not perceived (based on the median split)

In the group that did not perceive hubris, the results confirm a positive relationship between *benevolence* and *expected product success* (path coefficient = 0.27, *p* < = 0.01) and between *empathy* and the *expected product success* (path coefficient = 0.39, *p* < = 0.01). H4 and H5 are both supported. The relation between *expected product success* and *purchase intention* is supported when hubris is perceived (path coefficient = 0.67, *p* < = 0.001), and when hubris is not perceived (path coefficient = 0.75, *p* < = 0.001). According to Cohen (1988), the calculated effect sizes (*f*^*2*^) of H3 (0.57) and H6 (0.80 when hubris is perceived and 1.281 when hubris is not perceived) are considered large, while the effect sizes of H5 (0.15) are considered moderate. H1 (0.12) and H4 (0.12) are considered small (see Fig. 2 for more details).

### Post-hoc Multi-Group Analysis

Finally, we perform a PLS Multi-Group Analysis (MGA) — a nonparametric Mann–Whitney U test used to compare path coefficients between groups (Henseler et al., 2009). In this study, we use the method to compare the participants who perceived the entrepreneur in the pitch video as hubristic with those who did not; based on the median split. Table 2 summaries the results.

**Table 2:** PLS MGA results

Path coefficient/ adj. R^2^	Hubris not perceived (low median split group)	Hubris perceived (high median split group)	Difference significant?
AB** → **ES	−0.02	0.36*	**Yes**^**~**^
IN** → **ES	−0.09	−0.19	No
LE** → **ES	0.37**	0.67**	**Yes**^**~**^
BE** → **ES	0.27**	−0.13	Yes**
EM** → **ES	0.39**	0.03	Yes*
ES** → **PI	0.75***	0.67***	No
ES (adj. R^2^)	0.61	0.56	n.a.
PI (adj. R^2^)	0.56	0.43	n.a.

*AB* Ability, *BE* Benevolence, *ES* Expected product success, *EM* Empathy, *IN* Integrity, *LE* Legitimacy, *PI* Purchase intention

*p* < = 0.1 = ~; *p* < = 0.05 = *; *p* < = 0.01 = **; *p* < = 0.001 = ***

The results explain 56% of the variance in purchase intention (adjusted R^2^) in the group that did not perceive hubris and 43% of the variance in purchase intention in the group that perceived the entrepreneur as hubristic. Moreover, the results explain 61% of the variance in expected product success in the group that did not perceive hubris and 56% of the variance in purchase intention in the group that perceived the entrepreneur as hubristic.

The PLS MGA results suggest significant differences between individuals who perceive the entrepreneur in the crowdfunding video as hubristic and those who did not. The influence of benevolence differs with *p* < = 0.01 and the influence of empathy with *p* < = 0.05. The influence of ability and legitimacy differs with *p* < =0.1. When hubris is perceived, ability and legitimacy drive the expected product success. In contrast, when hubris is not perceived empathy and benevolence influence the expected product success. While legitimacy remains as a driver when hubris is not perceived, its influence is substantially lower compared to the case when hubris is perceived.

## Discussion

Founders that engage in forms of e-commerce as embodied in reward-based crowdfunding campaigns usually sell a vision of their future product to raise money to finance the turning of their prototypical products into reality. Primary empirical findings at the intersection of information systems and entrepreneurship literature indicate that the startup founders’ personality has a decisive influence on backers’ decision to purchase a future product in return for their investments (Ahlers et al., 2015; Allison et al., 2013; Moss et al., 2015), especially in absence of other reliable indicators (Sundermeier & Kummer, 2018). To shed light on the relevance of the entrepreneurial personality for the outcomes of crowdfunding campaigns, we focus on hubris, which is found to be particularly prevalent among startup founders (Bollaert & Petit, 2010; Hayward et al., 2006; Ranft & O’Neill, 2001).

We set out to answer the research question: *How does perceived hubris influence the trustworthiness in reward-based crowdfunding campaigns?* We find that expressions of hubris have a significant influence on the perception of startup founders’ trustworthiness that we assessed in terms of their ability, integrity, benevolence, empathy, and legitimacy. More specifically, we found that hubris signals strong ability and legitimacy to turn the planned product into a success, meet the stated delivery times, and fulfill the terms of the contract. However, as far as benevolence and empathy are concerned, their excessive pride and inflated feeling of self-worth are not appropriate signals of their receptiveness to customers’ wishes and needs, but rather the opposite. In summary, we find that both hubristic and non-hubristic entrepreneurs are successful in raising capital online. However, the underlying mechanisms differ, as hubristic entrepreneurs signal abilities and legitimacy, while the personality traits of non-hubristic founders signal benevolence and empathy.

### Theoretical contributions

Our study makes three main contributions to the literature on reward-based crowdfunding in particular and e-commerce in general, as well as to the wider field of entrepreneurial personality. First, we draw on theory in e-commerce literature to explain the inefficiencies in reward-based crowdfunding campaigns that account for the information asymmetries between startup founders and potential backers (Burtch et al., 2013; Kuppuswamy & Bayus, 2018). The popular option to pre-purchase a future product as a reward for an investment leads to the association of reward-based crowdfunding as a form of e-commerce (Ahlers et al., 2015; Beier & Wagner, 2015; Gierczak et al., 2014). However, existing theory is limited, as features that have been shown to be positively related to establishing buyers’ trust in regular online retailing transactions are seldom available for startup founders, who lack customer testimonials and a credible reputation, among other indicators (Bi et al., 2017; Burtch et al., 2013; Cholakova & Clarysse, 2015). In addressing this gap, we contribute to research that looks at the relevance of signals to crowdfunding campaigns’ outcomes (Hoegen et al., 2018; Koch & Siering, 2019). Previous findings indicate that the number of previous backers and their assessments of products and services, as shared through their social networks, are suitable signals of a campaign’s quality (Herzenstein et al., 2011; Lin et al., 2009; Thies et al., 2018; Zhang & Liu, 2012). However, these insights fail to explain how the network of supporters for a particular campaign grew in the first place. Therefore, scholars have started to assess the relevance of personality traits in these regards (Allison et al., 2015; Moss et al., 2015; Thies et al., 2016). Their contributions provide a valuable step forward in theory development but their focus on broad personality traits, like the ‘big 5′ model, replicates shortcomings that led management scholars to conclude two decades ago that “research on personality traits seems to have reached an empirical dead end” (Aldrich 1999, p. 76; Cogliser and Brigham 2004; Ensley et al. 2006; Rauch and Frese 2007). We address the limited predictive validity of these broader personality models by examining the implications of perceived hubris, a comparably narrow personality dimension that is particularly prevalent in the context of startups (Bollaert & Petit, 2010; Hayward et al., 2006; Ranft & O’Neill, 2001). Our findings suggest that potential backers pay close attention to how startup founders present themselves, as we find that signals of hubris have implications for backers’ assessments of a startup founder’s trustworthiness. Thus, we contribute first insights on signals that startup founders can use to transmit complex information regarding their motivations, skills, and capacities that will have a positive influence on potential backers’ expected product success and ultimately their funding intention.

Second, we add to the stream of research on e-commerce transactions, as our findings reinforce the necessity to shift the focus from a feature-oriented perspective to signaling perspective to capture holistically the determinants of perceived trustworthiness in these forms of online retailing transactions (Ahlers et al., 2015; Allison et al., 2013; Moss et al., 2015). While previous research determines the implications of certain features in the formation of trust (e.g., customer testimonials, direct communication channels, design, quality seals, and security measures), we extend research in this area by demonstrating that hubris can serve as a signal of a startup founder’s abilities integrity, and legitimacy. These insights are useful not only for reward-based crowdfunding campaigns but also for retailers who are involved in regular e-commerce transactions. For instance, retailers often use videos in their social media campaigns to attract customers. While the established features of these videos can help to generate trust for the purposes of a transaction, little is known about the signals that pique customers’ attention and curiosity in this competitive landscape. In this context, we provide novel insights because traditional theories such as the transmission model (Shannon, 1948) assume that all receivers interpret signals uniformly. We argue that this does not apply to personality traits such as hubris. Therefore, we build upon the semiotic theory that suggests that the perception of signals is highly individual. This allows us to distinguish two types of trust personas: Those who perceive hubris in the given context and those who do not. The findings suggest that these two groups exist and that different trust mechanisms influence their intention to back the crowdfunding campaign.

Third, the focus on hubris allows us to contribute to the hubris theory of entrepreneurship by suggesting that hubris is beneficial for the venture creation (Bollaert & Petit, 2010; Hayward et al., 2006; Ranft & O’Neill, 2001). Our findings show the influence of hubris on expected product success and ultimately the funding intention of potential backers. Despite hubris’ predominant label as a dark personality dimension (Judge et al., 2009; Owen & Davidson, 2009; Petit & Bollaert, 2012), our findings empirically confirm that perceptions of hubris positively affect the assessment of startup founders’ trustworthiness and suggest that (perceived) personality traits serve as a signal of founders’ abilities and legitimacy. These results are particularly useful because crowdfunders who pre-purchase future products are confronted with considerable information asymmetries, and their investments are subject to a high degree of risk (Agrawal et al. 2014). Crowdfunders’ rely on their perceptions of founders’ personality because all the information they have is the textual and visual descriptions of the (prototypical) product provided by the founders themselves. Especially early backers face these difficulties, as they can rely on neither objective external information nor previous backers’ assessments of the product as signals of quality (Thies et al., 2018; Zhang & Liu, 2012).

### Practical implications

Our results have implications for startup founders who initiate reward-based crowdfunding campaigns, potential backers who need to handle information asymmetries in assessing the trustworthiness of founders, and supporters of entrepreneurial endeavors, such as investors, consultants, and coaches. Our findings help to inform these parties about the power of perceived personality traits. Therefore, both parties can take advantage of our findings. First, backers of crowdfunding campaigns should be aware that unconscious factors can affect their funding decisions, so they should not lose sight of the associated risks.

However, second, startup founders can also use our results to influence potential backers’ assessments of their crowdfunding campaigns by displaying certain personality traits. Hubris is particularly likely to have positive implications for backers’ funding decisions when ability and legitimacy are particularly relevant. Existing research on charisma indicates that the ability to show certain personality traits can be taught (Antonakis et al., 2011). While previous research considers hubris to be a dark and, thus, unfavorable personality trait, hubristic entrepreneurs should not hide this facet of their personalities but display it openly in their video pitches. These insights are also of value to e-commerce providers who use videos to commercialize their products.

Third, educational institutions and startup coaches who prepare founders to raise money for their entrepreneurial endeavors could offer trainings related to our findings. These workshops could be particularly useful for women, who still face difficulties in raising money due to biases by investors (Brush et al., 2002; Kanze et al., 2018), as displaying certain traits that are normally attributed to men could have positive implications for how their trustworthiness is assessed. However, further research that examines the relationship between hubris and gender is required, as our results provide only a first step in the use of personality in videos used in crowdfunding and e-commerce. Nevertheless, offering such hubris training should include a discussion of the moral aspects of deliberately manipulating one’s personality to increase the likelihood of an investment decision.

## Conclusion

We examined how perceived hubris displayed by startup founders influence assessments of their trustworthiness in a reward-based crowdfunding scenario. We found that expressions of exaggerated self-confidence and inflated self-worth signal their abilities and legitimacy that positively influence the assessments of startup founders’ trustworthiness. Through this research, we contribute to broadening the scope of the literature on e-commerce transactions and emphasize the necessity of shifting the research foci from a feature-oriented perspective to a signaling perspective. In addition, we highlighted the relevance of examining the influence of perceptions of narrow personality traits on the success of reward-based crowdfunding campaigns. Despite having been carried out with much caution, our study has limitations that open avenues for future research. First, we limit our study to hubris, but there are other comparatively narrow traits, such as overconfidence and narcissism, that have also been shown to be relevant in the startup context. Further research is required to identify them and explore their effects. Second, as in most survey-based research, our sample size is too small to be representative of the general population. In addition, due to cultural heterogeneity in the country, the restriction of the sample to U.S. citizens does not preclude other influencing factors from playing a role in the evaluation of the displayed founder personality and the purchase decision. Future research may provide interesting contributions on how different cultural and experiential backgrounds influence the relationship between perceived hubris and the funding decision. Third, our results are limited as we used one actor to display hubris in relation to one fictitious product. While explorative in nature, our research provides impetus for future research on how the vendors personality affect online transactions and the underlying decision processes to back a crowdfunding campaign or purchase a product. We hope that future research emphasizes additional narrow traits that are related to the startup context to generate a more holistic understanding of the determinants of crowdfunding funding, especially in early phases of such campaigns, where the network effects from existing and future backers are absent.

## Appendix A. Measurement instrument

**Table 3:** Question items and related quality criteria

Construct (reference)	Factor loading	Composite reliability	AVE	Cronbach’s Alpha
	(low perceived hubris group / high perceived hubris group)			
Ability (Mayer & Davis, 1999)		0.927/0.954	0.720/0.806	0.901/0.940
AB1: The entrepreneur is very capable of performing his job.	0.726/ 0.886			
AB2: The entrepreneur has much knowledge about the work that needs to be done	0.885/ 0.923			
AB3: I feel very confident about the entrepreneur’s skills.	0.915/ 0.910			
AB4: The entrepreneur has specialized capabilities that can increase the performance of the product.	0.809/ 0.881			
AB5: The entrepreneur is well qualified	0.893/ 0.887			
Benevolence (Mayer & Davis, 1999)		0.969/ 0.984	0.939/ 0.968	0.935/ 0.967
BE1: The entrepreneur is very concerned about my welfare.	0.969/0.982			
BE2: My needs and desires are very important to the entrepreneur.	0.969/0.985			
Empathy (adapted from Plank et al., 1996)		0.914/ 0.893	0.780/ 0.736	0.860/0.824
EM1: I feel as if I am on the same wavelength as this entrepreneur.	0.932/0.896			
EM2: This entrepreneur does not understand how I think. (reversed)	0.893/0.830			
EM3: This entrepreneur has a lot of knowledge about how I need to make decisions.	0.822/0.847			
Expected product success (adapted from Chen et al., 2009)		0.902/ 0.917	0.754/ 0.787	0.835/ 0.863
ES1: The business model made sense.	0.806/0.823			
ES2: There is an attractive market for the product.	0.889/0.904			
ES3: The business idea is profitable.	0.907/ 0.931			
Integrity (Mayer & Davis, 1999)		0.890/ 0.925	0.733/ 0.804	0.811/ 0.878
IN1: I don’t have to wonder whether the entrepreneur will stick to his word.	0.711/ 0.845			
IN2: I like the entrepreneur’s values.	0.931/ 0.895			
IN3: Sound principles seem to guide the entrepreneur’s behavior.	0.909/ 0.948			
Legitimacy (Pollack et al., 2012)		0.948/ 0.953	0.902/ 0.910	0.892/ 0.902
LE1: I envision the business receiving high-profile endorsements in the future.	0.944/ 0.960			
LE2: I envision this business having a top management team that will benefit the organization.	0.955/ 0.949			
Purchase intention (Pavlou, 2003; van der Heijden et al., 2003)		0.967/ 0.988	0.935/ 0.975	0.931/ 0.975
PI1: Under the assumption that this would be an actual crowdfunding campaign, I intend to purchase the product.	0.971/0.987			
PI2: How likely is it that you would consider purchasing a product from this crowdfunding campaign?	0.963/ 0.988			

## Appendix B. Cross loadings

**Table 4:** Cross loadings between question items (low split group)

	Ability	Benevolence	Empathy	Expected product success	Integrity	Legitimacy	Purchase intention
AB1	0.726	0.385	0.570	0.444	0.618	0.537	0.478
AB2	0.885	0.238	0.651	0.480	0.596	0.603	0.524
AB3	0.915	0.489	0.764	0.590	0.782	0.705	0.666
AB4	0.809	0.372	0.467	0.360	0.412	0.448	0.378
AB5	0.893	0.514	0.615	0.581	0.564	0.664	0.480
BE1	0.439	0.969	0.469	0.585	0.568	0.589	0.598
BE2	0.491	0.969	0.458	0.587	0.507	0.516	0.459
EM2	0.703	0.468	0.932	0.743	0.663	0.678	0.768
EM3	0.707	0.433	0.893	0.579	0.661	0.672	0.710
EM4	0.519	0.355	0.822	0.500	0.537	0.516	0.516
ID1	0.623	0.549	0.588	0.806	0.483	0.612	0.591
ID4	0.463	0.469	0.628	0.889	0.500	0.632	0.700
ID5	0.466	0.560	0.613	0.907	0.523	0.637	0.658
IN1	0.417	0.466	0.467	0.415	0.711	0.430	0.419
IN2	0.722	0.547	0.689	0.525	0.931	0.676	0.531
IN3	0.664	0.419	0.638	0.536	0.909	0.665	0.527
LE1	0.682	0.546	0.697	0.646	0.716	0.944	0.695
LE2	0.669	0.538	0.655	0.722	0.619	0.955	0.658
PI1	0.568	0.519	0.732	0.766	0.493	0.682	0.971
PI2	0.607	0.538	0.750	0.678	0.635	0.695	0.963

**Table 5:** Cross loadings between question items (high split group)

	Ability	Benevolence	Empathy	Expected product success	Integrity	Legitimacy	Purchase intention
AB1	0.886	0.482	0.522	0.466	0.612	0.483	0.433
AB2	0.923	0.538	0.632	0.552	0.657	0.594	0.561
AB3	0.910	0.581	0.685	0.487	0.659	0.575	0.573
AB4	0.881	0.455	0.588	0.513	0.627	0.554	0.582
AB5	0.887	0.463	0.514	0.573	0.703	0.577	0.531
BE1	0.568	0.982	0.611	0.259	0.616	0.461	0.457
BE2	0.536	0.985	0.599	0.289	0.605	0.458	0.448
EM2	0.595	0.559	0.896	0.527	0.525	0.606	0.676
EM3	0.511	0.491	0.830	0.352	0.444	0.474	0.590
EM4	0.571	0.527	0.847	0.402	0.426	0.564	0.702
ID1	0.539	0.286	0.563	0.823	0.380	0.614	0.568
ID4	0.506	0.216	0.394	0.904	0.306	0.665	0.606
ID5	0.502	0.244	0.408	0.931	0.325	0.688	0.599
IN1	0.511	0.584	0.404	0.285	0.845	0.472	0.437
IN2	0.711	0.565	0.532	0.315	0.895	0.504	0.417
IN3	0.717	0.539	0.526	0.402	0.948	0.534	0.495
LE1	0.607	0.472	0.653	0.745	0.544	0.960	0.798
LE2	0.579	0.416	0.575	0.663	0.529	0.949	0.801
PI1	0.615	0.477	0.762	0.639	0.509	0.838	0.987
PI2	0.570	0.431	0.750	0.677	0.486	0.817	0.988

## Appendix C. Fornell-Larcker criterion

**Table 6:** Construct correlation and square root AVE score (low split group)

	Ability	Benevolence	Empathy	Expected product success	Integrity	Legitimacy	Purchase intention
Ability	0.849						
Benevolence	0.480	0.969					
Empathy	0.735	0.478	0.883				
Expected product success	0.593	0.605	0.702	0.868			
Integrity	0.714	0.554	0.706	0.578	0.856		
Legitimacy	0.711	0.570	0.710	0.722	0.700	0.950	
Purchase intention	0.606	0.545	0.765	0.749	0.579	0.711	0.967

**Table 7:** Construct correlation and square root AVE score (high split group)

	Ability	Benevolence	Empathy	Expected product success	Integrity	Legitimacy	Purchase intention
Ability	0.898						
Benevolence	0.560	0.984					
Empathy	0.654	0.615	0.858				
Expected product success	0.581	0.279	0.510	0.887			
Integrity	0.728	0.620	0.547	0.378	0.897		
Legitimacy	0.622	0.467	0.645	0.740	0.562	0.954	
Purchase intention	0.599	0.459	0.766	0.666	0.503	0.837	0.988

## Footnotes

1 https://www.kickstarter.com/projects/ably/ablytm-unbelievably-odor-and-stain-repellant-natur
